# The epidemiology, risk factors, and impact on hospital mortality of status epilepticus after subdural hematoma in the United States

**DOI:** 10.1186/2193-1801-3-332

**Published:** 2014-07-01

**Authors:** Ali Seifi, Ali Akbar Asadi-Pooya, Kevin Carr, Mitchell Maltenfort, Mehrdad Emami, Rodney Bell, Michael Moussouttas, Moussa Yazbeck, Fred Rincon

**Affiliations:** Department of Neurosurgery, Division of Neurocritical Care, University of Texas Health Science Center at San Antonio, Mail Code 7843, 7703 Floyd Curl Drive, Medical School Building 102F, San Antonio, TX 78229-3900 USA; Neurosciences Research Center, Shiraz University of Medical Sciences, Shiraz, Iran; Jefferson Comprehensive Epilepsy Center, Department of Neurology, Thomas Jefferson University, Philadelphia, USA; The Rothman Institute, Philadelphia, PA USA; Department of Neurology and Neurosurgery, Thomas Jefferson University, Philadelphia, USA; Division of Neuro Critical Care, Capital Institute for Neurosciences, Trenton, USA; Department of Neurosurgery, John Muir Medical Center, Walnut Creek, USA

**Keywords:** Subdural hematoma, Status epilepticus, Risk factors, Mortality

## Abstract

**Introduction:**

Subdural hematoma (SDH) is a well described risk factor in the development of Status Epilepticus (SE), however the epidemiology of SE after SDH is unknown. In this study, we sought to determine the epidemiology of SE, the prevalence of risk factors, and impact on hospital mortality using a large administrative dataset.

**Methods:**

Data was derived from the Nationwide Inpatient Sample from 1988 through 2011. We queried the NIS database for patients older than 18 years, with a diagnosis of SDH and SE. Diagnoses were defined by ICD 9 CM codes 432.1, 852.2, 852.3 and 345.3 for SE. Adjusted incidence rates of admission and prevalence proportions were calculated. Multivariate logistic models were then fitted to assess for the impact of status epilepticus on hospital mortality.

**Results:**

Over the 23-year period, we identified more than 1,583,255 admissions with a diagnosis of SDH. The prevalence of SE in this cohort was 0.5% (7,421 admissions). The population adjusted incidence rate of admissions of SDH increased from 13/100,000 in 1988 to 38/100,000 in 2011. The prevalence of SE in SDH, increased from 0.5% in 1988 to 0.7% in 2011. In hospital mortality of patients with SDH and without SE decreased from 17.9% to 10.3% while in hospital mortality of patients with SDH and SE did not statistically change. Mortality increased over the same period (2.3/100,000 in 1988 to 3.9/100.000 in 2011) and the diagnosis of SE increased mortality in this cohort (OR 2.17, p < 0.0001). The risk of SE remained stable throughout the study period, but was higher among older patients, blacks, and in those with respiratory, metabolic, hematological, and renal system dysfunction.

**Conclusion:**

Our study demonstrates that the incidence of admissions of SDH is increasing in the United States. Despite a decline in the overall SDH related mortality, SE increased the risk of in-hospital death in patients with a primary diagnosis of SDH.

## Introduction

Subdural hematomas (SDH) are most often described as resulting from traumatic brain injury (TBI) of varying degrees. In survivors of TBI, post traumatic epilepsy (PTE) is a well-known complication (Tomkins et al. 2008; Storti et al. 2012; Steg 1993; Hung and Chen 2012; Gupta and Gupta 2006; Agrawal et al. 2006), and often complicates treatment in the hospitalized patient (Annegers et al. 1998). The epidemiology of status epilepticus in patients admitted with SDH is however poorly understood. Similarly, the pathophysiology of epileptogenesis in these patients is incompletely known, but is thought to be biphasic, with 1) early and 2) late post traumatic epilepsy (PTE) (Agrawal et al. 2006). One hypothesis states that in the immediate peri-injury period, brain swelling, cerebral ischemia and the release of excitatory amino acids and other toxins precipitate neuronal damage (Agrawal et al. 2006; Brodersen and Gjerris 1975). Comparatively, in the late post injury period, an excess of extracorpuscular hemoglobin facilitates the creation of cytotoxic hydroxyls, reactive oxygen species moieties and glutamate accumulation (Payan et al. 1970). These compounds collectively promote neuronal damage propagating the creation of an epileptogenic nidus, and occasionally leading to status epilepticus (Willmore et al. 1978; Rubin and Willmore 1980; Shaver et al. 1996; Sahuquillo-Barris et al. 1988; Becker 1986).

The diagnosis of status epilepticus in the setting of an extra-axial hemorrhage in general portends a poor prognosis (Dennis et al. 2002; Claassen et al. 2006; Little et al. 2007). In patients with acute or chronic SDH the risk of developing epileptiform activity is higher and increases in those requiring surgical evacuation (Yeh et al. 2013; Englander et al. 2003). In addition to high mortality and morbidity, the diagnosis of SDH presents a significant economic burden on healthcare systems and over the last several decades has been correlated to increases in health care costs (Frontera et al. 2011; Balser et al. 2013). Although, surgical intervention through trephination or craniotomy assisted evacuation has improved mortality in some cohorts, seizures and the diagnosis of status epilepticus (SE) may complicate recovery (Annegers et al. 1998; Rubin and Rappaport 1993; Cameron 1978; Drapkin 1991; Kotwica and Brzezinski 1991; Robinson 1984; Cole and Spatz 1961; Huang et al. 2011; Byung-Soo et al. 2008).

However, the epidemiology of status epilepticus (SE) after SDH has not been studied despite the fact that an association between SDH and seizures is widely demonstrated in academic literature (Rubin and Rappaport 1993; Cole and Spatz 1961; Rabinstein et al. 2010; Sabo et al. 1995; Ohno et al. 1993). Similarly, the risk factors for SE in patients with SDH have not been sufficiently characterized in previous literature (Huang et al. 2011; Westmoreland 2001; Wiedemayer et al. 2002). The purpose of this study was to determine the epidemiology of SE in patients with SDH and to characterize the associated risk factors for SE in these patients. We also investigated the impact of SE on in hospital mortality in patients with SDH.

## Materials and methods

In this retrospective study, data was obtained from the Nationwide Inpatient Sample from 1988 through 2011. The NIS, a database maintained as part of the Healthcare Utilization Project of the Agency for Healthcare Quality and Research (HCUP-AHQR), is the largest all-player inpatient database representing an approximate stratified 20% sample of all non-federal, short-term, general, and specialty hospitals serving adults in the United States.

The International Classification of Disease – Clinical Modification, 9th revision (ICD-9-CM) codes used to identify SDH patients [ICD-9-CM codes 432.1 (non-traumatic SDH) and codes 852.2 or 852.3 (traumatic SDH)] as previously reviewed in the literature (Frontera et al. 2011). These codes had a 70% sensitivity, 100% specificity, 94% positive predictive value, and 100% negative predictive value when listed as the primary or secondary diagnoses (Frontera et al. 2011). This cohort included patients with acute, sub-acute and chronic SDH with the aforementioned admission ICD-9-CM codes Table 1.

**Table 1:** **The ICD-9-CM classification of study cohort**

ICD9-CM code	Description	References
**432.1, 852.2, 852.3**	Subdural-hematoma	(11, 17, 41, 43)
**518.5, 518.82**	Grand Mal Status	
**96.70, 96.71, 96.72**		
**458.0, 458.8, 458.9, 796.3, 785.51, 785.59**	Cardiovascular dysfunction, hypotension, shock	-27
**286.2, 286.6, 286.9, 287.3-5**	Hematological dysfunction, disseminated intravascular coagulation, purpura fulminans, coagulopathy, thrombocytopenia	-27
**570, 572.2, 573.3**	Hepatic dysfunction, acute hepatic failure, hepatic encephalopathy, hepatitis	-27
**584, 580, 585, 39.95**	Renal dysfunction, acute renal failure, acute glomerulonephritis, renal shutdown, hemodialysis	-27
**293, 348.1, 348.3, 780.01, 780.09, 89.14**	Neurological dysfunction, transient organic psychosis, anoxic brain injury, encephalopathy, coma. altered consciousness, electroencephalography	-27
	Comorbidities (Charlson et al. 1987)	(13, 27, 30)
**428.0-428.9**	Congestive heart failure	
**401**	Hypertension	
**249, 250**	Diabetes mellitus	
**491, 492, 496**	COPD	
**571**	Chronic liver failure	
**585, 586**	Chronic kidney disease	
**196, 199**	Cancer	

SDH, SE, co-morbidities, and acute organ dysfunctions used in this study.

We included patients 18 years of age or older, with a primary or secondary diagnosis of SDH. Patients’ age, gender, and race (white, African-American, Asian-Pacific Islander, American/Indian Eskimo, other; and not stated) were obtained from the NIS database. From the cohort of patients with SDH, those who developed SE during their hospital admission, were identified using ICD-9-CM code 345.3 (Claassen et al. 2007; Urtecho et al. 2013) as previously validated in the literature (Urtecho et al. 2013). A 2013 study by Urtecho and colleagues validated that the accuracy of the ICD-9-CM code for SE was 100% when defined as a neurologist’s documentation of continuous clinical seizure activity for five minutes or longer, and/or two or more discrete seizures without interictal return to baseline (clinical diagnosis) and/or EEG consistent with SE per a board-certified neurophysiologist’s interpretation (EEG diagnosis) (Urtecho et al. 2013). Based on clinical diagnosis alone, the ICD-9-CM code for SE carried a sensitivity of 82% (95% CI, 72–92%), specificity of 100%, PPV 100%, and NPV of 90% (95% CI, 83–98%); based on EEG data alone, the ICD-9-CM code for SE carried a sensitivity of 55% (95% CI, 42–67%), specificity of 100%, PPV of 100%, and NPV of 79% (95% CI, 69–89%) (Urtecho et al. 2013).

In-hospital complications and organ failures that might have been related to the primary diagnosis, were identified among all patients, using the same ICD-9-CM codes as demonstrated in previous literature (Charlson et al. 1987; Quan et al. 2005; Rincon et al. 2012). Hospitals were divided by hospital location into: urban, sub-urban, and rural; by type into community (non-academic), academic (university-based), and public; and according to the Halpern criteria (Halpern et al. 2006), into small to medium size (<300 beds), large (301-499 beds), and extra-large (>500 beds).

National estimates were calculated according to accepted guidelines for the accuracy of NIS-HCUP data (Cameron 1978). That is, using the provided discharge weights, accounting for stratification, and clustering effects. For trend analysis we used the provided supplemental files that incorporate the newly calculated discharge weights using the 1998 NIS definition for the discharge population and available from the NIS-HCUP website (http://www.hcup-us.ahrq.gov/db/nation/nis/nistrends.jsp). Continuous data were presented as means and standard deviations or medians and inter-quartile ranges (IQRs), as appropriate. Categorical data was presented as proportions and 95% confidence intervals (CIs). Odds ratios (Schulte et al. 2008) were calculated to determine independent predictors of hospital mortality. Given the dichotomous outcome, multiple logistic regression modeling was used. All factors of interest were included and parsimonious models were found by systematically removing the least significant factor and recalculating the model. The analysis was conducted using Structured Query Language (SQL) and the LME-4 package (ver. 0.99) in the R programming language for statistical computing (ver. 2.11), both available under the GNU Public License (http://cran.r-project.org/). Pearson chi-square test was used to determine statistical differences of corresponding demographic parameters between 1988 and 2011. P-values are two sided and statistical significance was judged when p < 0.05. Our reporting of observational data conforms with Strengthening the Reporting of Observational Studies in Epidemiology STROBE guidelines (von Elm et al. 2007). Based on the de-identified nature of the database, the study was exempted from full IRB review.

## Results

**Figure 1:**
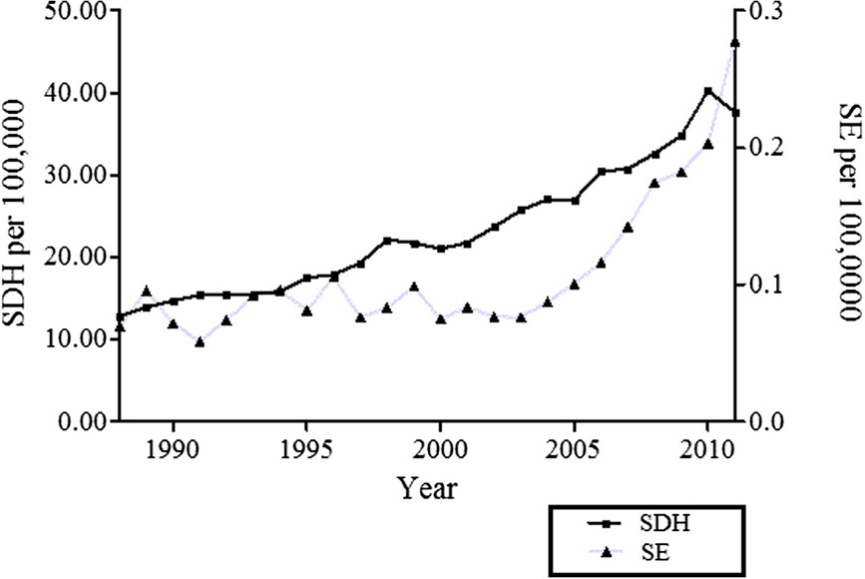
**The population adjusted rate of subdural hematoma (SDH) hospitalizations from 1988 through 2011 in the USA.**

Over the 23-year period, we identified 1,583,255 admissions with a diagnosis of SDH. The prevalence of SE in this cohort was 0.5% (7,421 admissions). More than 50% of admitted patients self-identified as white, with a male predominance of 59%, (Table 2). The population adjusted incidence rate of admissions of SDH increased from 13 per 100,000 in 1988 to 38 per 100,000 in 2011, with a concomitant increase in the prevalence of SE diagnosis in this cohort, (Figure 1). More than half of admitted patients were treated in private institutions, with relatively equal geographic representation. Black patients had a statistically significantly higher prevalence of SE that their white counterparts (OR 1.71, p < 0.001), (Table 3).

**Table 2:** **Demographic characteristics of the study cohort**

Variable	No status epilepticus	Percentage	With status epilepticus	Percentage
**Number of SDH patients**	1575834	99.50%	7421	0.5
**1° SDH/2° SDH**	1130031 / 445803	71.7%/ 28.3%	4337 / 3084	58.4%/ 41.6%
**Age [Mean + SD]**	69.64 + 18.07		65.53 + 17.1	
**Female/Male**	646092 / 929742	41%/ 59%	2930 / 4491	39.5% / 60.5%
Race*				
White	884516	56.10%	3760	50.70%
Black	127327	8.10%	1147	15.50%
Hispanic	95653	6.10%	581	7.80%
Asian	39711	2.50%	141	1.90%
Native American	6303	0.40%	59	0.80%
Other	30256	1.90%	159	2.10%
Not stated	391910	24.90%	1573	21.20%
Hospital characteristics*				
Rural	111254	7%	323	4.30%
Urban private	913669	58%	4405	59.40%
Urban academic	550912	35%	2693	36.30%
Northeast	307918	19.50%	1479	19.90%
Midwest	352829	22.40%	1523	20.50%
South	586368	37.20%	2758	37.20%
West	328561	20.90%	1660	22.40%
Small to medium (<300 beds)	111096	7%	470	6.40%
Large (300–500 beds)	330295	21%	1633	22.00%
Extra-large (>500 beds)	1134285	72%	5317	71.60%
**Major organ dysfunction**				
Respiratory	133631	8.50%	2701	36.40%
Hematological	92659	5.90%	804	10.80%
Hepatic	11188	0.70%	185	2.50%
Neurological	107472	6.80%	1492	20.10%
Metabolic	26632	1.69%	498	6.71%
Renal	137728	8.74%	1322	17.82%
Cardiovascular	55154	3.50%	555	7.48%
**Outcome**				
**In hospital mortality**	210531	13.40%	1937	26.10%

SDH: Sub-Dural Hematoma/*: Some Data is missing.

**Table 3:** **Predictors of status epilepticus among patients with subdural hematoma**

		95% CI	95% CI	
Variable	OR	(Lower)	(Upper)	P-Value
**Age (per year)**	0.99	0.99	0.99	<0.0001
**1° SDH vs. 2° SDH**	0.73	0.65	0.83	<0.0001
**Race (vs white)**				
Black	1.71	1.47	1.99	<0.0001
Hispanic	1.19	0.97	1.45	0.08
Asian	0.79	0.54	1.13	0.1
Native American	1.16	0.55	2.47	0.6
Other	1.04	0.72	1.5	0.8
**Hospital characteristics (vs small to medium)**				
Large	1.05	0.82	1.34	0.6
Extra-large	0.91	0.73	1.14	0.4
**Major organ dysfunction**				
Hematologic dysfunction	1.35	1.12	1.62	0.001
Metabolic dysfunction	1.86	1.48	2.35	<0.0001
Renal dysfunction	1.47	1.25	1.72	<0.0001
CNS dysfunction	2.26	1.95	2.62	<0.0001
Respiratory dysfunction	4.91	4.31	5.58	<0.0001

Vs. Versus, SDH: Sub-Dural Hematoma.

### Demographics

There were no statistically significant difference in the age distribution between patients admitted with diagnosed SE compared to those who did not in this study group (SE; 65.53 ± 17.1, no SE; 69.64 ± 18.07). The gender distribution was also similar in both the SE and non-SE cohorts, (39.48% and 41% female distribution respectively). The majority of patients admitted were identified as white (50.67% vs 56.13%), however among black patients there was a 50% greater prevalence of SE (Table 2).

### Morbidity and mortality

**Figure 2:**
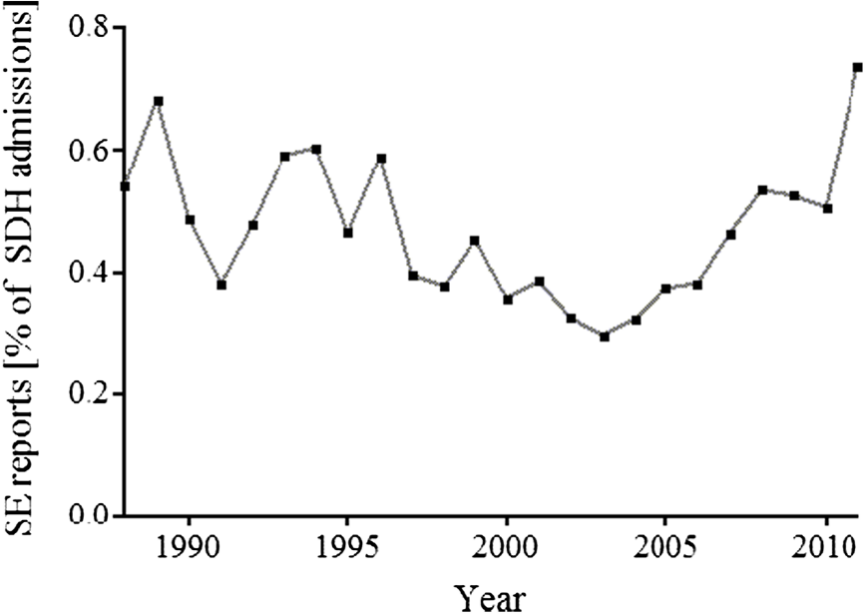
**Prevalence of status epilepticus in admissions with subdural hematoma (SDH) from 1988 through 2011 in the USA.**

**Figure 3:**
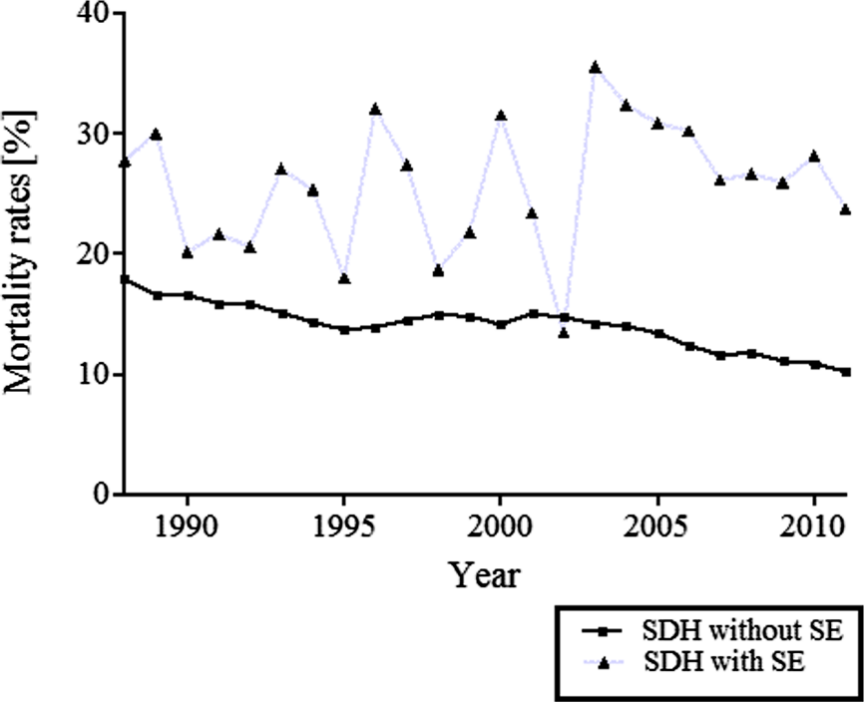
**Mortality rates among admissions with SDH, with and without status epilepticus from 1988 through 2011in the USA.**

Patients who presented with SE were more likely than not to have respiratory (36.4% vs 8.5%), hematological (10.8% vs 5.9%) or renal dysfunction (20.1% vs 6.8%), (Table 2). Respiratory dysfunction was the strongest predictor of SE in this cohort (OR: 4.91, p <0.0001). The prevalence of SE among patients with SDH increased nominally, from 0.5% in 1988 to 0.7% in 2011, (Figure 2); the diagnosis of SE in this sub-group was an independent predictor of in-hospital mortality (OR 2.17, p <0.0001), (Table 3). Over the course of study, there was a 7.6% reduction in the hospital mortality rate among patients with SDH without recorded SE, from 17.9% in 1988 to 10.3% in 2011, (Figure 3). Throughout the course of study, there was a 4.1% decrease in hospital mortality rates among patients with both SDH and SE, from 27.8% in 1988 to 23.7% in 2011 (Figure 3). Independent predictors of in-hospital mortality among admissions with SDH are shown, (Table 4).

**Table 4:** **Predictors of in-hospital mortality among patients with subdural hematoma**

		95% CI	95% CI	
Variable	OR	(upper)	(Lower)	P-Value
Status epilepticus	0.71	0.55	0.91	0.007
Primary SDH	1.06	1.03	2.95	<0.0001
Status epilepticus in 1° SDH	2.17	1.59	1.09	<0.0001
Age (per year)	1.01	1.01	1.1	<0.0001
Female (vs. male)	1.07	1.05	1.01	<0.0001
**Race**				
Black (vs. white)	0.83	0.8	1.01	<0.0001
Hispanic(vs. white)	0.83	0.79	1.07	<0.0001
Asian(vs. white)	0.88	0.82	0.95	0.001
Native American (vs. white)	0.88	0.73	0.87	0.2
Other (vs. white)	0.93	0.86	0.87	0.08
**Hospital Characteristics**				
Midwest (vs. Northeast)	0.91	0.87	0.88	<0.0001
South (vs. Northeast)	0.94	0.91	0.97	<0.0001
West (vs. Northeast)	0.84	0.81	0.95	<0.0001
Large (vs. small to medium)	1.18	1.11	1.16	<0.0001
Extra-large (vs. small to medium)	1.18	1.13	1.3	<0.0001
Rural (vs. academic)	1.23	1.16	1.24	<0.0001
Urban private (vs. academic)	1.13	1.1	1.24	<0.0001
**Major Organ Dysfunction**				
Cardiovascular dysfunction	2.35	2.23	2.47	<0.0001
Hematologic dysfunction	2.13	2.04	2.23	<0.0001
Metabolic dysfunction	1.86	1.73	2.01	<0.0001
Renal dysfunction	1.68	1.62	1.75	<0.0001
Hepatic dysfunction	1.64	1.46	5.18	<0.0001
CNS dysfunction	2.03	1.95	2.11	<0.0001
Respiratory dysfunction	5.01	4.85	1.85	<0.0001

SDH: Sub-Dural Hematoma, vs.: Versus.

## Discussion

In the cohort of patients admitted with a primary diagnosis of SDH, our assessment demonstrates an approximate three-fold increase in the incidence rate of admissions in the United States during the course of study. Similarly, while the prevalence of SE among admissions of SDH was low (0.5%) there was a statistically significant increase in incidence rate of admissions of SDH over the 23 years. Most importantly, though the diagnosis of SE in this sub-set was an independent predictor of mortality, (OR 2.17, p < 0.001) the in hospital mortality in the subset of patients with SE trended downwards over this time period but was not statistically significant. Comparatively, patients without SE demonstrated a statistically significant decrease in hospital mortality. Whether there was a significant mortality difference between these two sub-groups cannot be ascertained from current data.

**Figure 4:**
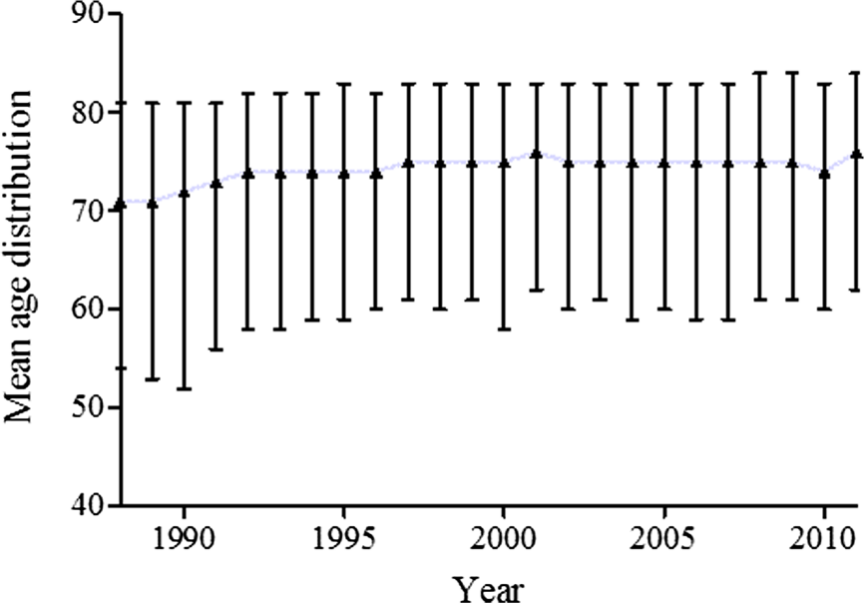
**Variance in the age distribution in subdural hematoma (SDH) hospitalizations from 1988 through 2011 in the USA.**

In 2011, a retrospective analysis of SDH diagnoses as reported by the NIS demonstrated similar trends in admissions. Frontera et al showed a 39% per capita increase in hospitalization for patients with SDH (Frontera et al. 2011). This increase in incidence has previously been attributed to an aging population in the USA (Frontera et al. 2011; Kudo et al. 1992; Santarius et al. 2009). However factors such as, improved diagnosis either due to physicians’ evolving index of suspicion, and more available computed tomography (CT) scanners cannot be ruled out (Smith-Bindman et al. 2009; MPAC (U.S.) 2007; Amis et al. 2007). Similarly, we demonstrate a three-fold increase in the primary diagnosis of SDH patients who are hospitalized in the United States, (Figure 1) with no significant variance in the age distribution, (Figure 4).

The association between SDH and seizures and SE is repeatedly referenced in academic literature (Annegers et al. 1998; Rubin and Rappaport 1993; Cameron 1978; Drapkin 1991; Kotwica and Brzezinski 1991; Robinson 1984; Cole and Spatz 1961; Huang et al. 2011; Westmoreland 2001; Wiedemayer et al. 2002). On the contrary, large scale epidemiological studies of SE in this cohort is scarce. In our analyses, the prevlence of SE among SDH patients was low (0.5%) with increasing prevalence over the course of study. It is possible that this may reflect an underdiagnoses in hospitalized patients either due to a low index of suspicion based on other clinical findings or insufficient use of EEG studies. Studies by two groups Rubin and Rappaport ( 1993) and Ohno et al ( 1993) demonstrated comparatively higher prevalence of seizures in their single institution cohorts; 5.6% and 2.3% respectively. More recently, a series of 100 patients with diagnosed SDH suggested an 11% rate in diagnosis of inpatient seizure events, while there were no reported cases of SE (Huang et al. 2011). In a 2011 study, investigators retrospectively identified patients admitted with SDH who received surgical evacuation and electroencephalography (EEG) analysis. While 87% of this cohort had recorded epileptiform discharges, there were no reported cases of SE (Rudzinski et al. 2011). Quantitative improvement as diagnosed by EEG studies however correlated positively with improved long term functional outcome.

There are only a few case reports documenting SE in patients with SDH (Jones et al. 1989). The reason for this lower prevalence of SE among patients with SDH may be multi-factorial. Patients with trauma significant enough to cause SDH with post traumatic SE may have a higher mortality. We are unable to ascertain this relationship from NIS data. Additionally, for the identification of SE in the setting of SDH there needs to be a high clinical suspicion and in instances where patients are comatose secondary to significant injury, we recommend the use of continuous EEG recordings for diagnosis. Though the most recent European Society of Intensive Care Medicine (ESICM) guidelines strongly suggest incorporation of EEG assessments in this patient population (Claassen et al. 2013), low subscribership will result in under diagnosis. The underlying theory for epileptogenicity is hinged on the principle that acute ischemia in addition to exposure to cytotoxic mediators is the basis for neuronal injury and subsequent excitability (Shaver et al. 1996; Sahuquillo-Barris et al. 1988; Becker 1986). It follows that patients with an intact pial-arachnoid layer are less likely to develop PTE and SE (Tomkins et al. 2008). Whether patients who develop SE after SDH present with more leptomeningeal disruption than those who do not warrants further study.

Self-identified black patients were more likely to have a diagnosis of SE in the setting of SDH than other ethnic groups, Table 2. Asians on the other hand had a lower risk of SE in our study group. In previous studies, it has also been observed that the prevalence of epilepsy and SE is generally higher in African-Americans than other ethnic groups in the United States (Haerer et al. 1986; Logroscino et al. 2005; DeLorenzo et al. 1995). The reasons behind this finding is not well understood and warrants further studies.

Major organ dysfunction was correlated to the diagnosis of SE. Patients with a diagnosis of SDH were most likely to have SE if they presented with respiratory, renal or neurological dysfunctions, (Table 2). Whether or not this observation is related to acute co-existing systemic injury is currently unknown. It is also possible that these factors directly and indirectly affect the cost of disposition in addition to discharge morbidity in these patients. In 2011, Frontera and colleagues demonstrated increased hospital costs in patients presenting with multi-system injuries (Frontera et al. 2011). Consequently the diagnosis and treatment of any systemic derangement in patients with SDH may impact functional outcome in these patients.

This is the first assessment of the morbidity and mortality attendant to patients co-diagnosed with SE and SDH from data report in the NIS database. We demonstrate that SE in patients with a diagnosis of SDH is associated with an increased risk of mortality (OR 2.17, p < 0.001); a finding consistent with smaller studies (Sabo et al. 1995; Sung and Chu 1989; Claassen et al. 2003). We are unable to assess the impact or surgical intervention in the subset of patients with surgically correctable lesions, or the severity of TBI in the subset who presented after acute brain injury due to limitations of the dataset. However, in a retrospective assessment of patients undergoing SDH clot evacuation, Rabinstein and colleagues demonstrated a high incidence of epileptiform activity characterized by EEG recordings, (25%); SE was not reported in any case. Interestingly however, in their study, the presence of epileptiform discharges on EEG recordings were independently associated with poor post-operative outcome, but not long term functional recovery (Rabinstein et al. 2010). It is important to note that in patients with epileptiform activity anti-epileptic treatment was initiated (Rabinstein et al. 2010). Future studies assessing the impact of surgical intervention and the severity of trauma on SE should be undertaken.

From a public health standpoint, in critically ill patients with severe TBI and SDH the diagnosis and treatment of SE via continuous EEG monitoring has a positive mortality benefit (Bleck 2012), and should be a fixture in the management of this patient population. The treatment of electrocorticographic abnormalities with prophylactic AEDs may also have clinical benefit in some subsets of this patient population. In a recently published retrospective study, researchers demonstrated clinical efficacy in using either leviteracetam (LVT) or phenytoin for seizure prophylaxis in patients with acute or subacute SDH. One salient differences between the two cohorts was increased side effects historically attributable to phenytoin usage, when compared to the cohort treated with LVT (Radic et al. 2014). Contrastingly, Rappaport in 1993 reported the absence of evidence for AED usage in patients presenting with non-alcoholic patients with chronic SDH (Rubin and Rappaport 1993).

### Limitations of the study

This study has limitations which implicitly affects its interpretation. ICD-9 codes have questionable accuracy, since they may change over time (Frontera et al. 2011; Claassen et al. 2007; Urtecho et al. 2013). Secondly, the ICD-9-CM does not allow for differentiation between acute, chronic and sub-acute SDH which have differing risk factors, morbidity and mortality profiles. This study is an observational study thus not as robust as prospective analyses; neither can we assume etiological or causal relationships between variables and outcomes. Additionally, the NIS is an inpatient database representing an approximate stratified 20% sample of all non-federal, short-term, general, and specialty hospitals serving adults in the United States. This may not be an accurate representation of national trends in instances of under-reporting or under-diagnosis. As highlighted in similar studies, the data recorded in the NIS database includes both clinical and electroencephalographic diagnoses of SE without clear differentiation. The absence of standardization in the diagnosis of SE, introduces observer biases that cannot be corrected for in this study (Urtecho et al. 2013). Surgical interventions such as burr-hole craniotomies or trephination have been shown to affect morbidity after SDH (Pahatouridis et al. 2013; Krupp and Jans 1995). These variables were not assessed in our study due to the limitations of the dataset but may impact overall mortality/morbidity rates. Long term outcomes secondary to the diagnosis of SDH or SE were not assessed in this study; therefore it can be argued that long outcomes after discharge may differ from our reported data. The NHDS does not allow evaluation of various other variables such as other comorbidities, rates of DNR orders, timing of therapeutics and time of onset. These variables may have a confounding impact on our data. There may be patients with prior diagnoses of seizure disorders represented in this dataset. Since the NIS describes comorbidities upon patient discharge it is impossible to differentiate new diagnoses of seizure disorders from pre-admission diagnoses.

## Conclusions

Our study demonstrates an approximate three-fold increase in the incidence of SDH during the study period in the United States of America; however from our data the incidence of SE among patients with SDH was relatively low, (0.5%). The risk of SE among patients with SDH increased with age, and higher in African-Americans and in those with respiratory, hematological, or renal dysfunction. Despite a decline in the overall SDH related mortality, SE increased the risk of in-hospital death in patients with primary SDH during the study period. Increased utilization of EEG studies may positively impact in-hospital mortality through improved diagnosis in this patient cohort.

## Authors’ information

Ali Seifi, M.D., FACP: Assistant Professor of Neurosurgery, Neuro Critical Care. University of Texas Health Science Center at San Antonio, USA. Ali Akbar Asadi-Pooya, M.D: Associate Professor of Epileptology. Neurosciences Research Center, Shiraz University of Medical Sciences, Shiraz, Iran. Kevin Carr, M.D: Neuorsurgery Resident. University of Texas Health Science Center at San Antonio, USA. Mitchell Maltenfort, Ph.D: Statistician. The Rothman Institute, Philadelphia, USA. Mehrdad Emami, M.D: General Physician. Shiraz University of Medical Sciences, Shiraz, Iran Rodney Bell, M.D: Professor of Neurology and Neurosurgery. Thomas Jefferson University, Philadelphia, USA. Michael Moussouttas, M.D: Clinical Neurointensivist. Capital Institute of Neurosciences, Trenton, USA. Moussa Yazbeck, M.D., FACP: Clinical Neurointensivist, Department of Neurosurgery, John Muir Medical Center, Walnut Creek, USA. Fred Rincon, M.D., FACP, FCCP, FCCM: Assistant Professor of Neurosurgery and Neurology. Thomas Jefferson University, Philadelphia, USA.

## Abbreviations

SE: Status epilepticus
SDH: Subdural hematoma
CT: Computed tomarized
NIS: Nationwide inpatient sample
PTE: Post traumatic epilepsy.
